# Holomics - a user-friendly R shiny application for multi-omics data integration and analysis

**DOI:** 10.1186/s12859-024-05719-4

**Published:** 2024-03-04

**Authors:** Katharina Munk, Daria Ilina, Lisa Ziemba, Günter Brader, Eva M. Molin

**Affiliations:** Center for Health & Bioresources, AIT Austrian Institute of Technology, Konrad-Lorenz-Straße 24, 3430 Tulln, Austria

**Keywords:** Multi-omics, Multivariate data analysis, Biomarker discovery, Data integration, R shiny application, Sugar beet, Storability

## Abstract

An organism’s observable traits, or phenotype, result from intricate interactions among genes, proteins, metabolites and the environment. External factors, such as associated microorganisms, along with biotic and abiotic stressors, can significantly impact this complex biological system, influencing processes like growth, development and productivity. A comprehensive analysis of the entire biological system and its interactions is thus crucial to identify key components that support adaptation to stressors and to discover biomarkers applicable in breeding programs or disease diagnostics. Since the genomics era, several other ’omics’ disciplines have emerged, and recent advances in high-throughput technologies have facilitated the generation of additional omics datasets. While traditionally analyzed individually, the last decade has seen an increase in multi-omics data integration and analysis strategies aimed at achieving a holistic understanding of interactions across different biological layers. Despite these advances, the analysis of multi-omics data is still challenging due to their scale, complexity, high dimensionality and multimodality. To address these challenges, a number of analytical tools and strategies have been developed, including clustering and differential equations, which require advanced knowledge in bioinformatics and statistics. Therefore, this study recognizes the need for user-friendly tools by introducing Holomics, an accessible and easy-to-use R shiny application with multi-omics functions tailored for scientists with limited bioinformatics knowledge. Holomics provides a well-defined workflow, starting with the upload and pre-filtering of single-omics data, which are then further refined by single-omics analysis focusing on key features. Subsequently, these reduced datasets are subjected to multi-omics analyses to unveil correlations between 2-n datasets. This paper concludes with a real-world case study where microbiomics, transcriptomics and metabolomics data from previous studies that elucidate factors associated with improved sugar beet storability are integrated using Holomics. The results are discussed in the context of the biological background, underscoring the importance of multi-omics insights. This example not only highlights the versatility of Holomics in handling different types of omics data, but also validates its consistency by reproducing findings from preceding single-omics studies.

**Supplementary Information:**

The online version contains supplementary material available at 10.1186/s12859-024-05719-4.

## Background

An organism’s phenotype and vitality are the results of complex interactions between its genes, proteins, metabolites, many other molecular components, its microbes and its environment. In particular, external factors, such as biotic and abiotic stressors, can influence this balanced biological system dramatically, affecting growth, development and productivity. Therefore, analyzing such a biological system in its entirety with its interactions between different functional layers is crucial for i) identifying key components that can support adaptation to these stressors to maintain or even increase the vitality of an organism, or ii) discovering biomarkers that can be applied in plant and animal breeding programs, or for disease diagnostics and forecasting [1–4].

Since the era of genomics that began around 1990 [5], a large number of additional ’omics’ have emerged to this end: transcriptomics, metabolomics, microbiomics, proteomics, epigenomics, to name a few. In each of these fields, recent advances in high-throughput technologies have enabled the generation of large and complex datasets harboring a wealth of information about biological molecules and their interactions at that specific omics-level. These omics-levels are still traditionally analyzed individually, however, for the last decade, an increasing number of omics datasets have been analyzed in an integrative manner to gain additional information. This integrative approach, known as multi-omics analysis, has become more popular as high-throughput techniques are becoming increasingly cheaper. The multi-omics approach aims to gain a more holistic and systems-level understanding of the relationships and interactions between different biological components located across multiple layers of a biological system [6, 7].

Already, single-omics data are often of a large scale and complex structure requiring specialized analytical tools plus a certain knowledge base in bioinformatics. Multi-omics analysis is particularly challenging due to the high dimensionality of individual single-omics data, as well as the heterogeneity and multimodality of the overall multi-omics dataset, making the integration of these diverse datasets from multiple and heterogeneous sources (or modalities) into a meaningful model and the extraction of relevant information a formidable task. A variety of methods and strategies have been developed in recent years, ranging from clustering methods to co-expression to differential equations and modeling, and recently, also going into the sphere of machine learning; however, there is a need to expand the knowledge of bioinformatics and statistics along this axis, as comprehensively reviewed [8, 9]. In addition, there is already a plethora of tools and packages for analyzing and integrating omics data [10]. However, many of them come with certain restrictions and limitations, e.g., they are tailored to a specific omics method, limited in the number of omics datasets or limited with regard to the species of interest. A very sophisticated and quite user-friendly tool (through its well-established tutorials, webinars and workshops) that must be mentioned here is the R package mixOmics [11], which is making use of a multi-block data design and its integrative analysis are based on sparse multivariate models [12]. It is well used in the research community working on multi-omics data integration across multiple disciplines [13–15], it appears in multi-omics data integration guidelines and protocols [16, 17], and because it is designed to work seamlessly with other R packages and tools, many of mixOmics’ functions have been successfully implemented in various other packages, workflows, and pipelines, e.g., multiomics [18]. However, despite the availability of educational tools for learning multi-omics data integration approaches, using the necessary tools and packages, including mixOmics, often requires not only a deep understanding of statistics, but also good programming skills in R and/or Python, which may cause difficulties for some users. Therefore, the availability and development of tools should also focus on ensuring user-friendliness, especially for bioinformatics beginners, to be able to perform their first steps in multi-omics data integration.

A number of user-friendly, web-based tools are already available to the community that do not require advanced programming skills. For example, MetaboAnalyst can process raw input data from both targeted and untargeted metabolomics. It offers integrated pathway analysis of genes and metabolites, but only works with transcriptomics and metabolomics data [19]. PaintOmics 4 provides a graphic interface and utilizes biological pathway maps to analyze and visualize multi-omics datasets focusing on the combination of metabolomics with transcriptomics and/or proteomics data [20]. On the other hand, 3Omics implements a correlation network-based approach with a simple and clear interface, but is currently only suitable for analyzing human data and is limited to transcriptomics, proteomics, and metabolomics data [21]. Web-based platforms that explicitly mention also the inclusion of microbiomics data are, for example, OmicsNet 2.0 [22] and MiBiOmics [23]. The first uses a network-based multi-omics approach and the latter incorporates weighted-gene correlation network analysis, is implemented as an easy-to-use R shiny application, but is currently limited to a maximum of three omics datasets. Other applications based on R shiny [24] that address multi-omics data integration and analysis include FORALL, tailored for acute lymphoblastic leukemia cell lines [25], GMIEC, tailored for human data [26], ShinyOmics, mainly designed for the downstream analysis of transcriptomics data [27], or an yet unpublished application for multi-omics analysis of inflammatory bowel disease [28].

In order to provide a tool that is not limited to any organism or number of omics datasets, and in particular to address beginners in multi-omics data analysis, we developed Holomics, an easy-to-use R shiny application with a selected set of multi-omics functions mainly based on the R package mixOmics [11]. One of the novelties of Holomics lies in the implementation of an automated filtering process to reduce high dimensional input datasets, which is based on the median absolute deviation (MAD). Furthermore, the mixOmics-based tuning procedures are automatized in Holomics. Specifically, when there are feature columns with a near-zero variance usually causing algorithm failure, the datasets are automatically adapted and the tuning process is restarted without any necessary interaction by the user. In addition, Holomics offers the possibility to explore the calculated associations between the omics datasets through an interactive network [29]. And last but not least, some Holomics plots allow for a custom color scheme defined by the user.

## Implementation

Holomics was implemented in R version 4.2.0 [30] using the R package shiny [24] to make it an easy-to-use, interactive web application. Most of the integrated analysis algorithms rely on the R package mixOmics [11]. In detail, we integrated the by mixOmics developed mixMC framework [31, 32] for preprocessing microbiomics datasets, as well as the single-omics analyses (s)PCA (with its functions pca and spca) and (s)PLS-DA (with the functions plsda and splsda). The sparse version of the analyses is used during the tuning procedures. In addition, Holomics integrates mixOmics’ pairwise-omics analysis (s)PLS (functions pls and spls), and its multi-omics analysis DIABLO (with the function block.splsda) in its supervised version.

**Fig. 1:**
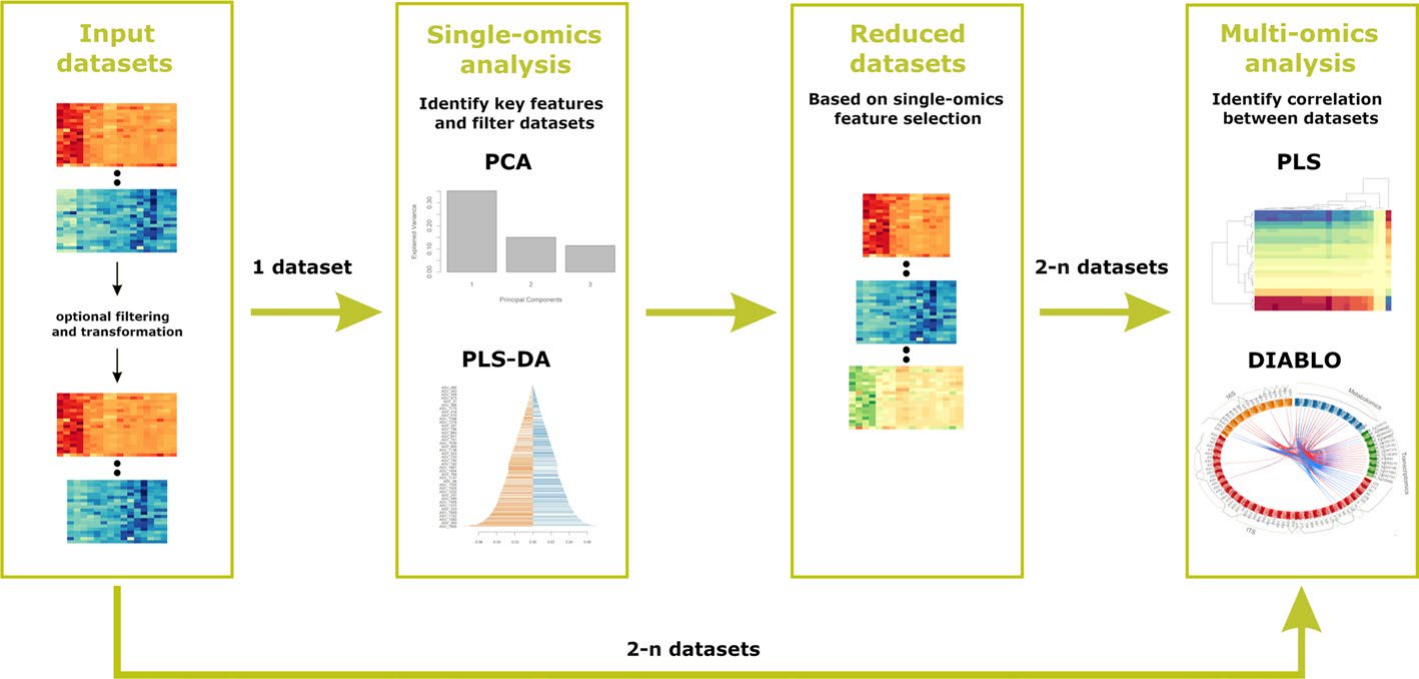
The Holomics workflow. To make use of all the functionality provided by Holomics, a certain workflow should be followed. (1) Input datasets: first, the datasets are uploaded where an eventual pre-filtering/transformation step takes place. (2) Single-omics analysis: afterwards, the single-omics analysis is performed, where key features are identified and the datasets are reduced accordingly. (3) Reduced datasets: the single-omics feature selection process is resulting in reduced datasets. (4) Multi-omics analysis: with these reduced datasets (or with the input datasets from step 1), the multi-omics analyses are applied to identify correlations between 2-n datasets

When using Holomics, a three-step workflow as shown in Fig. 1 is recommended: first, data are uploaded (Sects. “Input data” and “Data upload”); second, single-omics analysis including feature reduction is performed (Sect. “Single-omics analyses”); and third, multi-omics analysis is done (Sect. “Multi-omics analyses”). A more detailed description is given in the Holomics vignette [33].

### Input data

When working with omics data and prior to any integrative analyses, the user needs to tackle difficulties such as class imbalance, missing data, data heterogeneity, the curse of dimensionality, and diverse scalability problems [34]. Also in case of Holomics, a certain preprocessing of the datasets might need to be performed before their upload, e.g. in case of missing data, an imputation task has to be done. There are different strategies and tools to impute omics datasets, e.g. for metabolomics data MICE [35] or for transcriptomics and microbiomics data the R package missForest [36] can be recommended. Alternatively, eUTOPIA [37] allows its users to preprocess any microarray data.

In general, any kind of omics data on a continuous scale can be used for the integrative analysis of Holomics. However, sequence-based count data needs to be processed before using Holomics to convert it to continuous data. To further improve the results from multi-omics data integration, users need to address the remaining, above mentioned difficulties separately as Holomics lacks any further built-in normalization or class balancing algorithms. Mirza et al. [34] provide a good overview on how and with which tools the data preparation tasks can be tackled.

Examples of omics data that can be used are a (FPKM-, TMM-, etc. [38]) normalized read count table as a transcriptomics dataset or normalized quantities of the measured metabolites as a metabolomics dataset [39]. Microbiomics, at its simplest level, investigates the composition of microbial communities, which is typically done using 16S rRNA or ITS profiling, for bacterial or fungal communities, respectively [40]. The microbiomics dataset(s) can therefore consist of a table of amplicon sequence variants (ASVs or OTUs, operational taxonomic units). Beyond these examples, also proteomics, phenomics, metagenomics, metatranscriptomics, etc. data can be included, as long as they are continuously scaled, pre-processed and normalized. Examples of metabolomics, microbiomics, and transcriptomics data as well as a file with the labels and class information can be found in Additional file 1: Tables S1﻿–S5. These omics data serve as test datasets for Holomics and can be uploaded directly into the application after removing the first line with the table title in each case. In addition, exactly the same datasets were processed with Holomics in the herein described case study.

### Data upload

Basically an unlimited number of datasets from any omics kind can be uploaded, whereas every upload file can have a maximum size of 50 Mb. As mentioned before, Holomics does not provide any omics-specific normalization algorithms. Only in the case of microbiomics data (e.g., in form of an ASV table), which have to be specified as such during data upload, the mixMC framework [31, 32] is applied in the background as a pre-processing step. If the omics dataset contains more than 10,000 features, Holomics automatically filters the dataset to 10,000 or fewer features. Therefore, firstly, low count filtering is used, meaning that all feature columns with a column sum less than 10 are removed, and secondly, if necessary, the remaining n columns with the lowest MAD are removed to obtain the maximum of 10,000 features. The whole filtering process is performed because it is recommended to use the mixOmics algorithms with a maximum of 10,000 features per dataset [11].

In addition to the omics datasets, at least one corresponding metadata file needs to be uploaded, which contains the labels or class information of the samples in the corresponding omics data. Furthermore, the metadata file can include a personalized color scheme for the distinct classes that are used later in the plots.

### Single-omics analyses

Single-omics analysis in Holomics can be performed either using the unsupervised principal component analysis (PCA) [41] or the supervised partial least squares discriminant analysis (PLS-DA) [42]. When following the Holomics workflow (Fig. 1), the key concept of single-omics analysis is to identify the key features of each omics layer and to reduce the dataset accordingly for further usage in the multi-omics analyses.

With regard to PCA, this filtering process calculates the number of components that are needed to obtain at least 80% of the explained variance. Afterwards, sparse PCA (sPCA) [43] is used to determine the information-rich features of the before calculated components. In the end, the dataset is reduced to only these important features. Compared to that, the filtering process of the PLS-DA takes the number of the pre-selected components, puts it into a sparse PLS-DA (sPLS-DA) model [44], where the number of features per component is tuned using n-fold cross-validation, and finally takes the number of components and associated features that have the lowest balanced error rate (BER) during the tuning process. Also here, the reduced dataset consists only of the above mentioned associated features. In both cases, the resulting feature-selected dataset is automatically available for the subsequent multi-omics analyses and can additionally be downloaded manually.

Generally, both single-omics pages present several plots visualizing the results of the uploaded as well as the reduced filtered dataset next to each other. These plots include a sample plot, which is a visual representations of the sample similarities, and a number of variable plots showing the influence of the features on the selected or calculated number of components.

### Multi-omics analyses

#### Pairwise-omics analysis

**Fig. 2:**
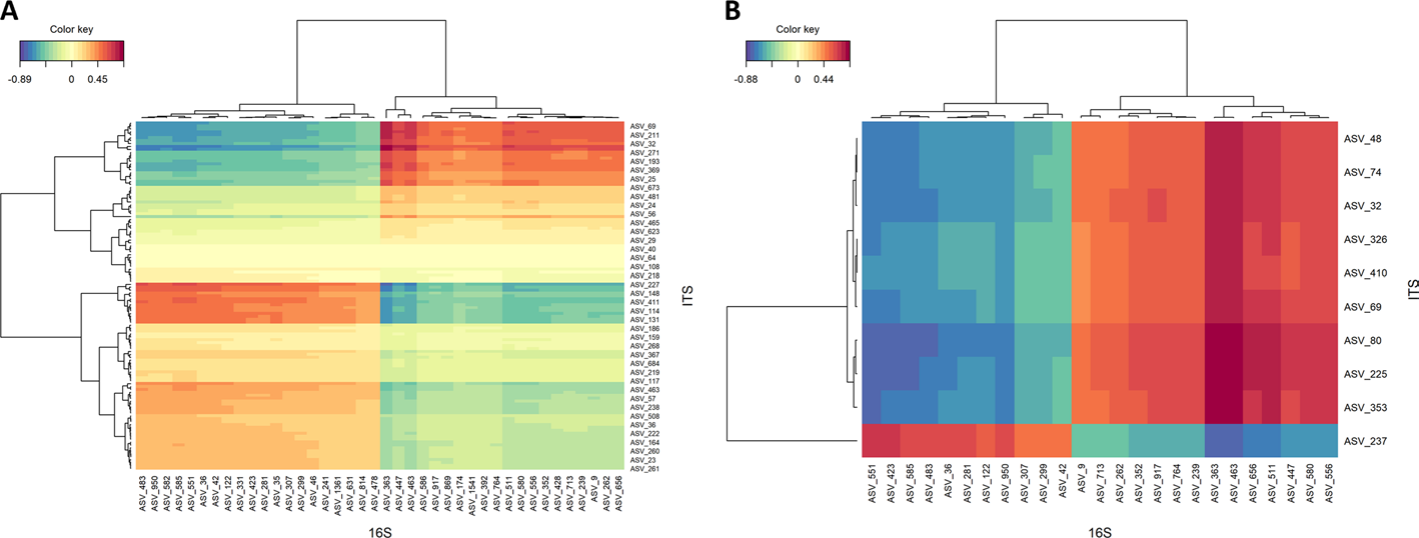
Example of the sPLS tuning effect using heatmaps. (s)PLS analysis and the tuning process were performed with two microbiomics datasets (ITS and 16S). **A** Result of the (s)PLS analysis using the two mixMC pre-processed, PLS-DA-filtered and within the PLS analysis standardized datasets, ITS (119 features) as dataset X of the analysis and 16S (40 features) as dataset Y. The analysis was performed using canonical mode and four components. The heatmap visualizes the correlations between the features of the two datasets. **B** Result of the (s)PLS analysis after the tuning process, which reduced the ITS dataset down to 10 features and the 16S dataset to 25 features. Additionally, the ideal number of components is 1. The heatmap shows the correlations between the features of the two reduced datasets. Note: Feature names were removed from the heatmaps

One of the multi-omics analyses integrated in Holomics is the unsupervised, pairwise, multivariate version of the sparse partial least squares s(PLS) analysis [45], which can be used to analyze two omics datasets (X and Y). In general, mixOmics provides multiple modes for the (s)PLS algorithm, whereas in Holomics only the regression and canonical mode can be used. When using the regression mode, the algorithm tries to predict dataset Y using dataset X, so changing the order of the datasets leads to different results. On the other hand, with the canonical mode the datasets should be interchangeable, and this mode is especially relevant when there is no prior known dependency between the two datasets [46]. However, during our case study, we observed that interchanging the datasets, when using the canonical mode, still led to different results. Like in the feature selection process of the single-omics analyses, multi-omics analyses include a tuning process. The tuning process of the (s)PLS analysis takes the number of pre-selected components and calculates the $Q^2$ score per component using n-fold cross-validation. During this calculation the algorithm can fail due to feature columns, which have a variance that is near zero. If the algorithm fails, the tuning process of Holomics calculates the percentage of distinct values per feature column for both datasets. It then determines which dataset has the column(s) with the lowest uniqueness percentage and removes these column(s) from the determined dataset. Afterwards, the calculation of the $Q^2$ score gets restarted. This whole procedure is performed as long until the calculation algorithm finishes or one or both datasets become too small. In general, the tuning process determines the correlation between the actual and predicted components using different configurations for the number of features selected per dataset. In the end, the last number of components with a total $Q^2$ above 0.0975 [47] is the ideal number of components and the number of features, which had the highest correlation, is the ideal number of features for the datasets used. Based on this information, the dataset is tuned down to only contain the selected features and is subsequently used to recalculate the (s)PLS analyses. Within the Holomics application, the user can always see the result plots of the (s)PLS using the untuned datasets on the left side of the page and the plots using the tuned datasets on the right side. Figure 2 shows the effect of the (s)PLS tuning process using two microbiomics datasets. It is clearly visible that the tuning algorithm cuts out the features of both datasets with a positive or negative correlation between the datasets.

#### Multi-omics analysis

**Fig. 3:**
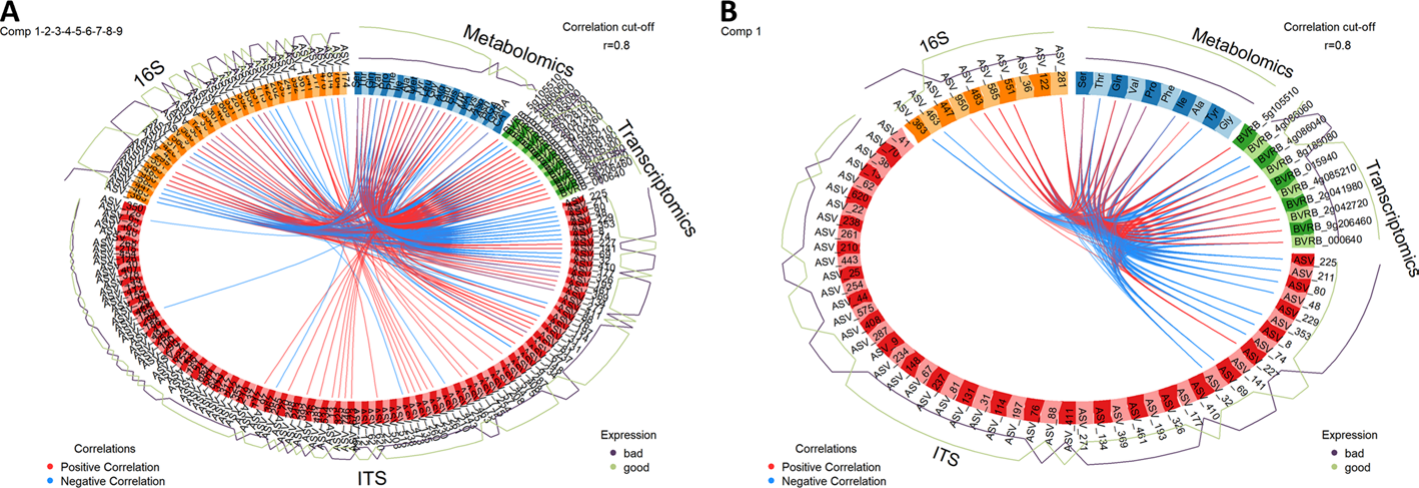
Showcase of DIABLO tuning effect using Circos plots. Usage of the DIABLO analysis and tuning process with a metabolomics, a transcriptomics and two microbiomics datasets (16S and ITS). **A** Result of the DIABLO analysis using the four untuned, PLS-DA-filtered and within the DIABLO analyzes standardized datasets, metabolomics (23 features), transcriptomics (16 features), 16S (40 features) and ITS (119 features), and nine components. The Circos plot shows the correlations between the features of the four untuned datasets using an absolute cutoff value of 0.8. **B** Result of the DIABLO analysis after the tuning process, which reduced the metabolomics dataset to 10 features, transcriptomics to 10 features, 16S to 10 features and ITS to 50 features. Additionally, the ideal number of components is 1. The Circos plot shows the correlations between the features of the four reduced datasets using an absolute cutoff value of 0.8

The last analysis integrated in the Holomics workflow is the multiblock sPLS-DA multi-omics analysis (referred to as DIABLO, Data Integration Analysis for Biomarker discovery using Latent variable approaches for Omics studies [12]). To reach this step is the end-target of every Holomics user, as it maximizes the correlated information between the datasets and simultaneously identifies the key variables of the omics datasets. Generally, DIABLO can be used in a supervised and unsupervised fashion, whereas Holomics currently offers only the supervised version. When using the DIABLO analysis a design matrix must be specified, whereas the selection of the matrix can be determined based on a variety of aspects. One of them is a data-driven aspect, where the value of the design matrix is based on the pairwise correlations, calculated with the PLS analysis, of the provided datasets [12]. Holomics calculates the pairwise correlations automatically as soon as the user selects the datasets, which should be used for the DIABLO analysis. The lowest calculated correlation is then automatically set for the design matrix. However, the value is always adjustable by the user as e.g. there could be a prior known biological correlation between the datasets that should be used for the design matrix. Like for the (s)PLS analysis, a tuning process can be used to optimize the datasets for the DIABLO analysis. Similar to the tuning of the (s)PLS analysis, the DIABLO tuning process takes the user pre-selected components and fits a DIABLO model up to the number of components using n-fold cross-validation without any feature selection. During this calculation, similar to the (s)PLS algorithm, the DIABLO algorithm can fail due to feature columns, which have a variance that is near zero. If the algorithm fails, the tuning process of Holomics determines the datasets that led to the failure, calculates the percentage of distinct values per feature column for these datasets, determines the lowest percentage value and removes the column(s) with this uniqueness percentage value. Afterwards, the tuning process is restarted. This process is performed as long as any dataset leads to a failure of the process or until the calculation process is finished. In the end, the number of components is chosen based on the overall BER using the centroids distance metric. To obtain the number of features per dataset, n-fold cross-validation is performed using again the centroids distance metric. According to the determined parameters, the used datasets are tuned to contain only the necessary features. DIABLO analysis is performed again and the results are presented next to those of the original datasets. Figure 3 shows how the tuning process affects the number of features per dataset used for the DIABLO analysis.

### Plots

All the above mentioned analyses provide a number of plots to visualize the results. These include sample plots that visualize the samples of the datasets and variable plots that illustrate the connection between variables and components or the process by which the components are created from the initial variables [11]. One of the main graphs of the DIABLO analysis is the ’Relevance Network Graph’, which visualizes the correlations between the features of the different datasets in the form of a network [48]. Within Holomics, this graph is illustrated in an interactive way using the R package visNetwork [29]. First, the user can change the cutoff value to show only the connections and corresponding nodes for which the absolute correlation value is higher than the cutoff value. Second, the user can select a target node in the graph, resulting in the highlighting of the selected node along with its connected nodes, providing a clearer visualization of the interconnections between nodes. Finally, the nodes can be dragged around in the plotting area to change the structure of the graph and obtain a better overview.

## Results and discussion

To demonstrate the applicability and functionality of Holomics, we decided to address the described problem of post-harvest storability in sugar beet: after harvest, sugar beet is often stored for several weeks before it is processed. During this time, the sucrose is converted into invert sugars and mold forms, which leads to an economic loss for the entire sugar production and processing sector [49, 50]. Therefore, deciphering key factors associated with good storability is crucial because these factors can be used as biomarkers, e.g., to screen available varieties or to optimize breeding programs by including marker-assisted selection (MAS) targeting prolonged storability. Several single-omics studies have already been performed to address the above described problems [51–53]. However, a multi-omics analysis targeting this research question has not yet been performed.

### Case study datasets

**Fig. 4:**
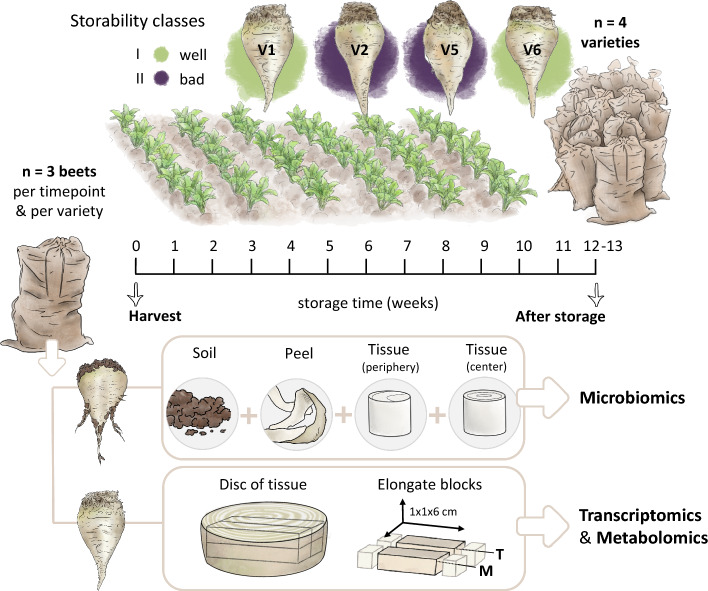
Sampling scheme of the case study input datasets. The following four varieties were included: two (V1, V6) with good storability (less sucrose loss, marked in green) and two (V2, V5) with increased sucrose loss after storage (purple coloring). After harvest, the sugar beets were stored in a semi-controlled environment for 12–13 weeks as previously described [51]. From three individuals per variety, samples for microbiomics (16S rRNA and ITS amplicon sequencing) were taken from the adhering soil, the peel, the tissue at the periphery and the tissue of the center of the beet root. For transcriptomic (T) and metabolomic (M) analyses, a disc was cut from the beet root, from which blocks were extracted and of which the outer first centimeter was removed [51]. Designed by Tatjana Hirschmugl

An overview of the analyzed sugar beet varieties, their storage behavior and which samples were taken for further omics-analysis is given in Fig. 4.

In detail, the transcriptomics data used in the present study include the count data of samples taken after 13 weeks of storage that were generated by Madritsch et al. [51]. Using the same beet root samples, targeted metabolomics (predominantly amino acids) was performed [52], representing the metabolomics input dataset. Although six sugar beet varieties with contrasting storage properties were analyzed in these two studies, we selected only the two varieties classified as well storable and the two least (badly) storable varieties for use in our case study. In addition, 16S rRNA and ITS amplicon sequencing for microbial community analysis was done on the same four varieties, but on different individuals which were stored for 12 weeks [54]. For microbiomics analysis, sampling was done on separate levels (soil, peel, peripheral tissue and inner tissue of the beet root); however, for this case study, these four levels were merged to reduce the analytical complexity. We followed a standard bioinformatics workflow as described [55]. The resulting ASV table represents the microbiomics input datasets in this case study. Each variety is represented by three biological replicates, summing up to a final dataset of 12 samples. The data tables used can be found in Additional file 1: Tables S1﻿–S5.

### Single-omics

For the integrative analysis, the original, unfiltered and normalized datasets were uploaded to Holomics. The two microbiomics datasets were automatically subjected to the mixMC pipeline and the transcriptomics dataset was filtered down to 10,000 features, as it originally exceeded this limit. Afterwards, each pre-filtered dataset was put into both single-omics analysis for feature selection, before going into the multi-omics analysis. The filtering process of the PLS-DA was performed multiple times with different settings for the pre-selected number of components (ranging from 3 to 7), whereas the received results did not change after a certain number of pre-selected components. Therefore, the last run, whose results were used for the following analyses, was performed with a number of pre-selected components that was in the middle of the testing range. Table 1 presents the number of features extracted during the upload, pre-filtering and single-omics filtering step. In general, compared to the PLS-DA, PCA tended to form a greater number of components during the filtering step. Also, the datasets shrank relatively less when using PCA.

**Table 1:** Summary of received results from pre-filtering and single-omics filtering processes

	M		16S		ITS		T	
Original no. features	23		4,398		3,252		27,964	
No. features after pre-filtering	–		779		290		10,000	
Single-omics analyses	PCA	PLS-DA	PCA	PLS-DA	PCA	PLS-DA	PCA	PLS-DA
No. features after filtering	23	23	232	40	252	119	112	16
No. pre-selected components	–	5	–	3	–	3	–	5
Ideal no. components	3	4	8	1	8	2	5	1
Filtering runtime (min)	1	2	10	10	10	10	5	15

Summary of the results obtained from the pre-filtering and single-omics filtering processes of the investigated omics datasets, metabolomics (M), bacterial microbiomics (16S), fungal microbiomics (ITS), and transcriptomics (T)

**Fig. 5:**
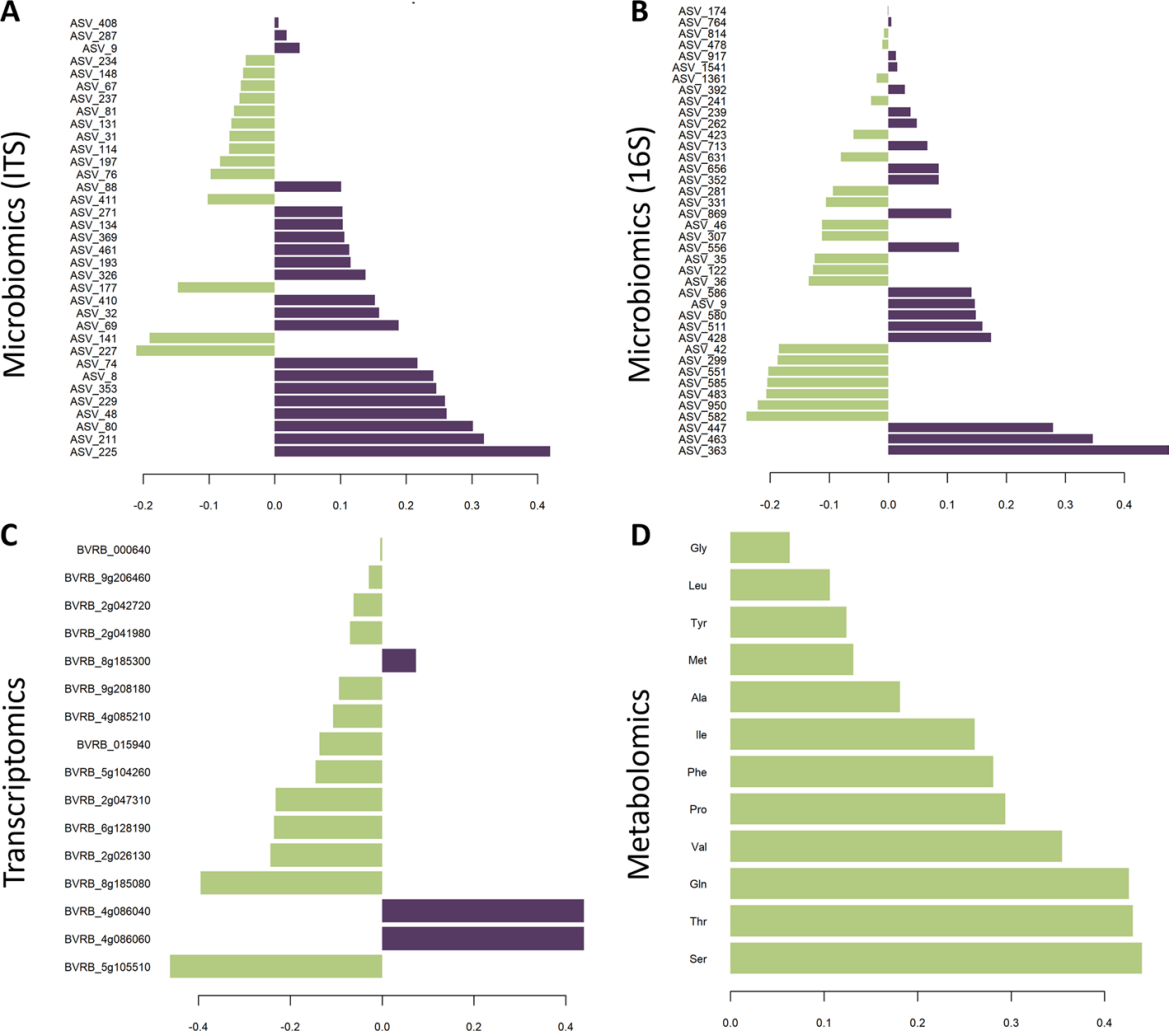
Contribution of component one for each of the single-omics datasets (**A**–**C**) after PLS-DA filtering. Green-colored features were associated with good storability, and purple-colored features were associated with bad storability

Following the guidelines of mixOmics [56], for interpretation of single-omics analysis and subsequent analysis steps, we focused on the results of PLS-DA, as this is the recommended single-omics method for a classification problem, as we have here in our case study. After the tuning/filtering process, one component was retained in case of transcriptomics and 16S data, two for ITS data, and four components for metabolomics data. In Fig. 5, the loading plots for the first component of each single-omics analysis are presented.

In the case of the targeted metabolomics analysis, all 12 amino acids within the first component showed association to good storability. This finding is in line with Gippert et al. [52], describing that the content of 15 out of 22 tested free amino acids was greater in the good storable sugar beet varieties than in the bad storable ones considering the time point after storage. Within the first component of the transcriptomics data, three out of 16 transcripts were association with bad storability: BVRB_8g185300, BVRB_4g086040 and BVRB_4g086060. All three transcripts also appeared to be significantly down-regulated at the last time point (after storage) in the badly storable varieties in Madritsch et al. [51], with log2-fold changes of $-$2.04, $-$3.72, and $-$4.22, respectively. Out of the 13 transcripts linked to good storability, five appeared to be in the above mentioned study among the significantly upregulated genes: BVRB_5g105510, BVRB_015940, BVRB_9g206460, BVRB_000640, and BVRB_2g026130, with log2-fold change values between 1.65 and 3.06, respectively. Single-microbiomics revealed 14 fungal ASVs (as proxies for taxa) associated with good storable varieties, 21 taxa associated with badly storable varieties (Fig. 5A), and 21 bacterial taxa associated to good and 19 taxa to badly storable varieties (Fig. 5B). A more detailed description of the storability-associated microbes is given in Wöber et al. [54].

### Pairwise omics

After performing the single-omics analyses, the filtered datasets were pairwise analyzed using (s)PLS. In this case study, one pair was formed by the two microbiomics datasets, 16S and ITS, and the second pair included the metabolomics and transcriptomics datasets. For both pairs, every dataset was once used as dataset X and once as dataset Y (they were analyzed bidirectionally) for the (s)PLS analysis. Additionally, the analyses were performed using once the PCA-filtered datasets and once the PLS-DA-filtered ones. All eight analyses were performed in the canonical mode, as we expected no prior known dependency between the datasets. In Table 2, the parameter settings used to obtain the final tuned datasets and the number of features of the datasets are summarized. Again, the tuning process was performed multiple times, using different numbers of the pre-selected components (ranging from 3 to 7); however, as the results did not change with an increasing number of pre-selected components, the final run was performed with only four pre-selected components. When using the canonical mode, the datasets should then be interchangeable without a change of the the tuning results [46]. But, in our case, different results for the number of features of the respective tuned datasets were returned.

**Table 2:** Summary of used configurations and received results testing the (s)PLS analyses

	Bidirectional (s)PLS			
Datasets	16S & ITS vs. ITS & 16S		M & T vs. T & M	
Filtered with	PCA	PLS-DA	PCA	PLS-DA
No. features after tuning	10 & 10 vs. 10 & 10	40 & 10 vs. 10 & 25	16 & 10 vs. 10 & 18	10 & 10 vs. 10 & 10
No. pre-selected components	4	4	4	4
Ideal no. components	1	1	1	1
Tuning runtime (min)	10	2	2	1

(s)PLS was performed with two omics dataset pairs. One pair was formed from the two microbiomics datasets (16S and ITS), and the second was composed of the transcriptomic (T) and metabolomic (M) datasets. Both pairwise analyses were done bidirectionally

**Fig. 6:**
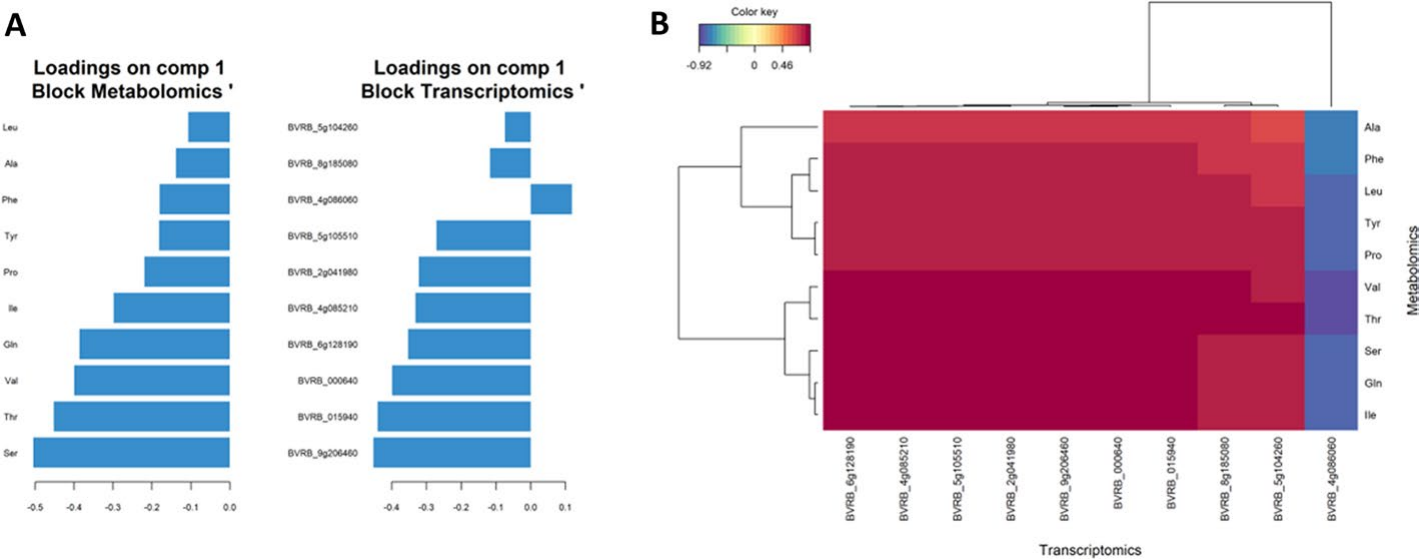
Loading plots (**A**) for metabolomics (left) and transcriptomics (right) and a heatmap (**B**) of pairwise analysis explaining the abundance of metabolites via transcriptomics

For the pairwise-omics analysis, we also focused on the interpretation of the results from PLS-DA (because of the classification problem we have, see above). For this case study, we first explained the expression of the metabolites with the transcripts (Fig. 6). The loading plot for the metabolites (Fig. 6A, left) resembles that of the single-omics analysis (cf. Figure 5D), where all of the metabolites show an association with good storability. In case of the transcriptomics data (Fig. 6A, right), ten transcripts appeared to be the major loadings on component 1, and among them, only BVRB_4g086060 was associated with bad storability (which was also seen in the single-omics analysis; cf. Figure 5C). Further, the heatmap (Fig. 6B) indicates a very similar pattern between the remaining nine transcripts and the ten metabolites. Interchanging the two datasets (to explain the transcriptomics data with the metabolites) did not lead to a change in the results.

**Fig. 7:**
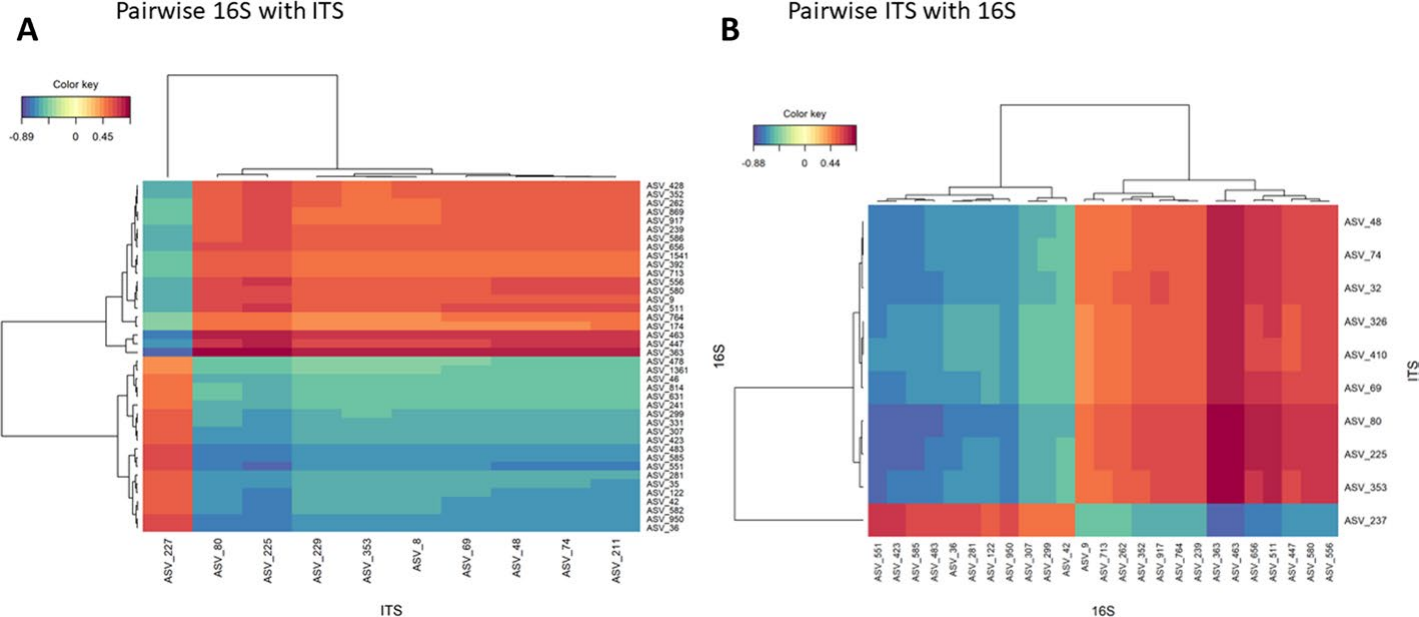
Heatmaps of pairwise microbiomics analysis. First analysis (**A**) explained bacterial microbiomics (16S dataset) with fungal microbiomics (ITS dataset), and the second analysis was done vice-versa (**B**)

Another pairwise analysis was performed with the microbiomics datasets. In this case, the results differed when the datasets were interchanged (Fig. 7). Explaining the bacterial communities (16S) with the fungal communities (ITS) led to ten fungal and 40 bacterial taxa (Fig. 7A). Here, one ITS taxon (ASV_227) had a different abundance than all the other ITS-based taxa. Among the 40 bacterial taxa (16S), the differential pattern appeared to be fifty-fifty. On the other hand, interchanging both datasets and explaining fungal communities (ITS) with bacteria led as well to ten (partly different) fungal, but only to 25 bacterial taxa (Fig. 7B). The latter form a subset of the 40 bacterial taxa found in the vice-versa analysis above. Here, one fungal taxon (ASV_237) exhibited a different pattern than all other ITS-based ASVs; however, this taxon was different from that detected via the vice-versa analysis.

Thus, deciding which dataset is the target dataset and which is the explanatory dataset is recommended based on the individual research question, eventually including a priori biological knowledge.

### Multi-omics

Finally, the DIABLO analysis was performed on all four omics datasets, once on the PCA-filtered ones and once on the PLS-DA-filtered. The analysis was only performed once with a rather high number of seven pre-selected components. The design matrix value was set based on the calculated pairwise correlation values: the lowest correlation value was rounded down to the tenth digit and set as the design matrix value. The parameters and sizes of the datasets are summarized in Table 3.

**Table 3:** Summary of the configurations and results from the DIABLO analysis

	DIABLO	
Datasets	M & T & ITS & 16S	
Filtered with	PCA	PLS-DA
No. features after tuning	10 & 10 & 10 & 14	10 & 10 & 50 & 10
Design matrix value	0.9	0.8
No. pre-selected components	7	7
Ideal no. components	1	1
Runtime (min)	120	40

For this multi-omics analysis, all datasets were integrated: the metabolomics (**M**), transcriptomics (**T**), and the two microbiomics (**16S** and **ITS**) datasets

**Fig. 8:**
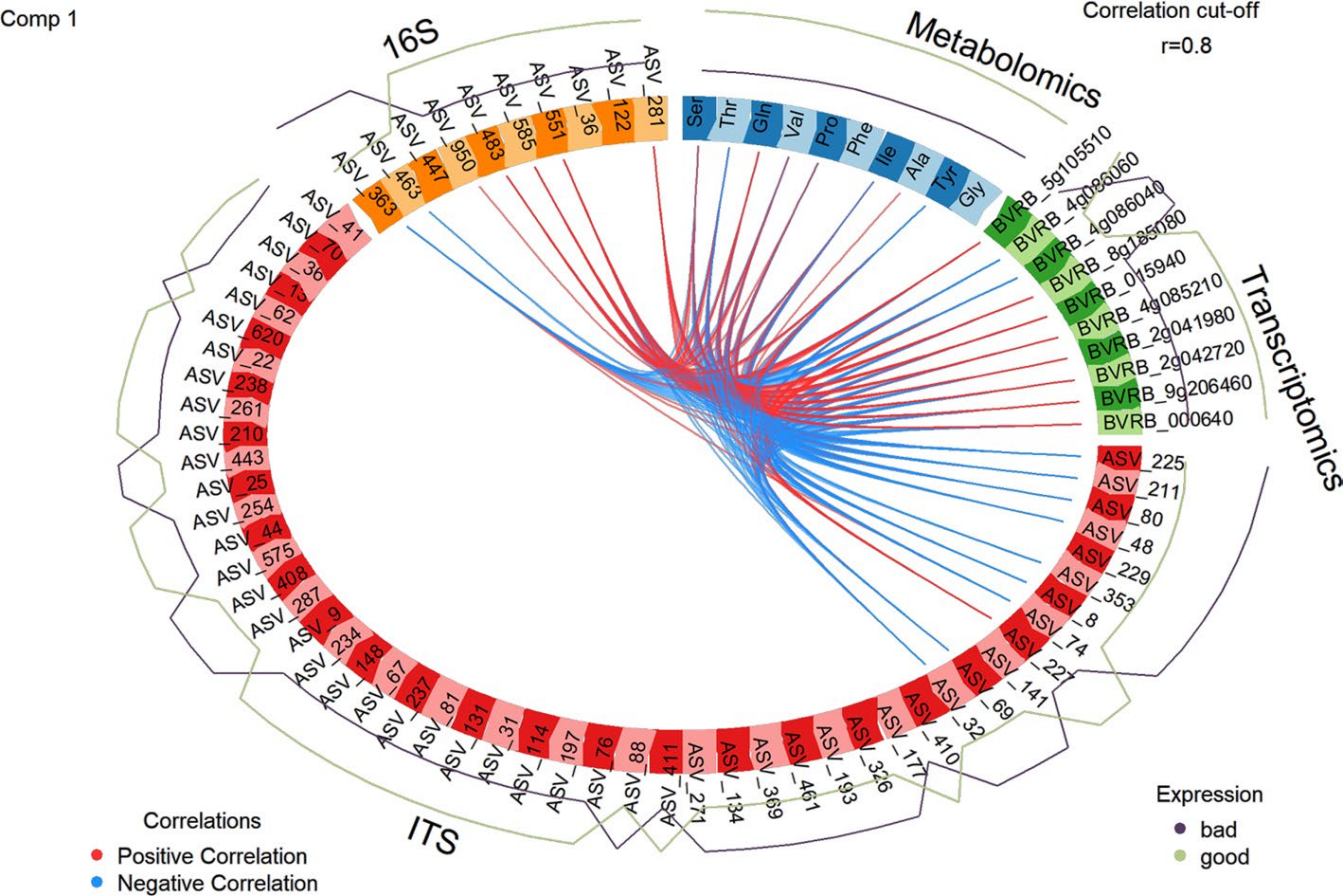
Circos plot from the DIABLO analysis indicating the positive and negative correlations among the features of all four omics datasets in the first component. For each feature, its ’expression’ with regard to the storability is presented as a continuous line: the green-colored line reflects the abundance of the feature in well storable varieties, whereas the purple-colored line shows the abundance in the badly storable ones

Also here, we focused on the description and interpretation of the results from the PLS-DA-filtered data (because of the classification problem we have, see above). In this case, the design matrix value was set to 0.8, as was the Circos plot correlation cutoff value. After tuning, seven bacterial taxa, ten fungal taxa, eight metabolites, and ten transcripts were correlated with each other above the 0.8 cutoff value (Fig. 8).

### Biological interpretation of the multi-omics analysis

Focusing on the metabolites first, among the free amino acids, threonine (Thr) showed the strongest (both negative and positive) correlations with the other omics datasets. Thr had strong positive correlations with several features that were well present in the well storable varieties, such as a bacterial taxon from the genus *Streptomyces* (ASV_551, 16S dataset) and one from *Nocardioides* (ASV_950, 16S dataset), both of which belong to Actinobacteria. Furthermore, Thr was also positively correlated with transcripts encoding the peptide methionine sulfoxide reductase (MSR) A1 gene (BRVB_5g105510), the mitochondrial frataxin (BRVB_2g041980) and mitochondrial superoxide dismutase (BRVB_2g042720). Notably, many features of the fungal ITS dataset showed negative correlations with Thr, the most strongly negative correlations with *Starmerella bacillaris* (ASV_225) and *Pichia membranifaciens* (ASV_80), which appear to be present in badly storable varieties. The non-Saccharomyces yeast *Starmerella bacillaris* (ASV_225) also exhibited strong negative correlations with all transcriptomics features. It is used in wine production because of its fermentation property [57, 58]. As it feeds on sugar, its presence most likely negatively influences the storability of sugar beet. At the same time, numerous species related to the genus *Streptomyces* have shown antifungal properties due to the production of antibiotics [59]. A *Streptomyces* strain was even described to inhibit the growth of *Starmerella bombicola* [60], providing support for the interaction found in our case study.

As mentioned above, Thr was among the amino acids found to be most abundant in the well storable varieties after harvest [52]. One explanation for the observed Thr-microbe interaction found in the well storable sugar beet varieties could be that some microbes living in symbiosis with a plant use its amino acids as a nutrient source. Plants can also convert amino acids into metabolites, which can later be used by microbes [61]. It is possible that Thr, among the other amino acids, supports the growth of a microbiome that positively affects the storability of sugar beet. It was found that Thr accumulation was connected with suppressed activity of the pathogen *Hyaloperonospora arabidopsidis* [62], which is an obligate biotrophic oomycete and a natural pathogen of the model plant *Arabidopsis thaliana* [63].

Concerning the identified correlated transcripts, methionine sulfoxide reductase A (MSRA, BRVB_5g105510) not only emerged in our case study in both single- and multi-omics analyses, but was also among the significantly upregulated genes documented in the well-storable varieties, as detailed in Madritsch et al.’s single-omics study [51]. MSRA is described to act as an antioxidant repair enzyme: the oxidation of sulfur-containing methionine in proteins inactivates these proteins, and MSRA repairs the damage by catalyzing the reduction of methionine sulfoxide back into methionine [64]. MSRA was reported to be an important oxidative stress resistance agent in *Corynebacterium glutamicum*. Without the activity of this gene, the studied bacteria exhibited a decreased cell viability, increased reactive oxygen species (ROS) production and increased protein carbonylation levels under various stress conditions [65]. A similar pattern has been described for plants, where MSRA expression levels are greater in plants under (photo)oxidative and osmotic stress conditions [66], and MSRA plays a key role in preserving the viability and life expectancy of an organism [67]. In addition, an increase in MSRA was also detected after infection with a virulent pathogen in *A. thaliana* [68], indicating its role in plant immune responses, as is described for MSRB [69]. Another gene, frataxin (BRVB_2g041980), was highly expressed in the well storable varieties and, as mentioned above, was positively correlated with Thr. This gene has been proven to be a mitochondrial iron-binding protein [70]. For plants, a balanced amount of iron is crucial for growth and development, as it activates essential metabolic pathways and is a component of many enzymes [71]. The main source of iron lies in the rhizosphere; however, a significant portion of iron is unavailable to the plant. A study showed that root microbes can mobilize this iron and make it accessible for the plant’s metabolism [72]. Interestingly, *Streptomyces* sp., which were positively correlated with frataxin in this study, were one of such microbes. These bacteria can produce siderophores, which are small-molecule metal chelators that support iron capture and transport under low-iron conditions [73–75]; conditions that also might be present during sugar beet storage.

In summary, this comprehensive multi-omics analysis revealed features associated with either well or bad storability of sugar beet, while detecting significant associations among these features. The elevated levels of free amino acids observed in well storable varieties may attract potentially beneficial microbes capable of producing antifungal agents, thereby suppressing fermenting yeasts and contributing to the observed improved storability. Additionally, methionine sulfoxide reductase A (MSRA) has been identified consistently as an upregulated gene in well storable varieties across both preceding single-omics analysis [51] and the herein conducted multi-omics analyses, affirming the robustness of the applied multi-omics methodology. Overall, this case study provides first valuable insights into the intricate interplay between metabolites, transcripts and microbial communities, shedding light on potential mechanisms across different omics layers influencing sugar beet storability.

## Runtime

For all three omics analyses the runtime of the filtering and tuning steps was measured at the minute level and documented in Tables 1, 2 and 3. As expected, the filtering process of the three feature-heavy datasets (both microbiomics and transcriptomics) had a longer runtime than that of the targeted metabolomics dataset with only 23 features. In contrast, the tuning processes differed greatly in terms of runtime, although the dataset sizes were similar. The analyses were performed and measured on a Lenovo Thinkbook with an 11th Gen Intel(R) Core(TM) i7–11,800 H processor and 32 Gb of RAM.

## Conclusion

**Fig. 9:**
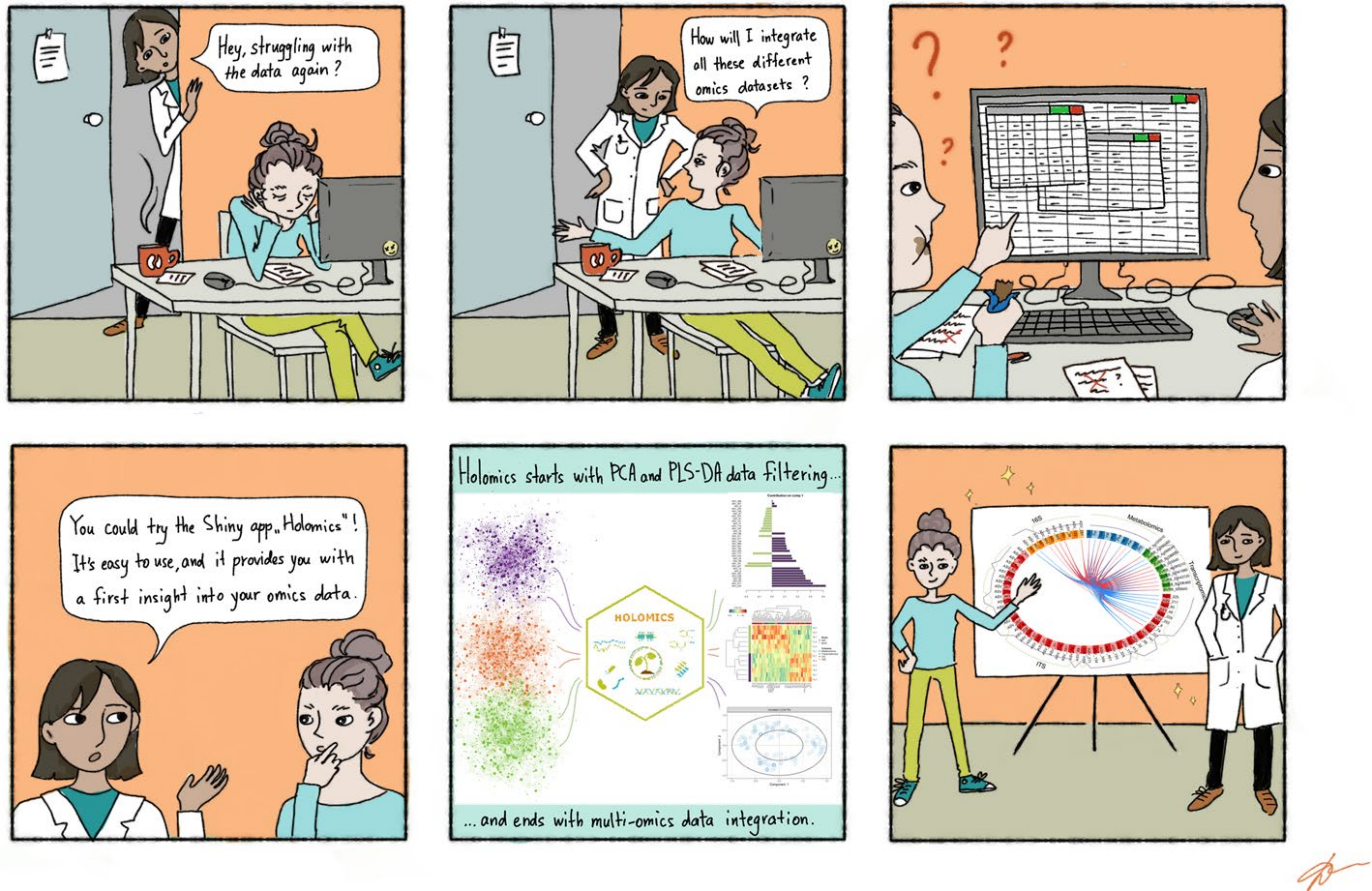
The R shiny application Holomics offers an easy-to-use and practical solution for multi-omics data integration and analysis

Holomics, an R shiny application, offers a practical and user-friendly solution for conducting multi-omics data integration and analysis (Fig. 9). Designed with an accessible interface and a guided workflow, Holomics is perfectly suited for researchers with limited bioinformatics knowledge or hardware resources. In the included case study, we applied Holomics to seamlessly integrate microbiomics, transcriptomics and metabolomics datasets from earlier single-omics studies elucidating factors, which are associated with improved storability. This practical demonstration not only highlights the application’s versatility in handling diverse data types, but also validates its consistency by reproducing findings from these preceding studies. In essence, Holomics simplifies omics analyses without compromising sophistication, making it an accessible resource for researchers seeking a practical and reliable tool for first insights into multi-omics investigations.

### Supplementary Information

**Additional file 1**. The additional file (Additional file 1.zip) is a compressed folder containing four .csv files. **Table S1**: Targeted metabolite data, **Table S2**: Microbiomics ASV count table resulting from 16S amplicon sequencing, **Table S3**: Microbiomics ASV count table resulting from ITS amplicon sequencing, **Table S4**: Transcriptomics read count table (transposed format), and **Table S5**: Labels and class information including color code of the analyzed samples. Besides of being the data source for the present case study, these data tables can be used as test dataset after removal of the table header (first line). We highly recommend opening the files in a text editor of your choice and remove the headers there. When doing this step in Excel an error may occur.

## Data Availability

Project name: Holomics. Project home page: https://cran.r-project.org/web/packages/Holomics/index.html  and https://github.com/MolinLab/Holomics. Operating system(s): Platform independent. Programming language: R. License: GNU GPL 3.0. Any restrictions to use by non-academics: No restrictions. The resulting omics data tables that were used in the herein described case study are available within the paper and its Supplementary information. In addition, the transcriptomics data are described in more detail in Madritsch et al. [51], metabolomics data in Gippert et al. [52] and microbiomics data in Wöber et al. [54], and can be accessed through information given in these publications.
