# Correction: Holomics ‑ a user‑friendly R shiny application for multi‑omics data integration and analysis

**DOI:** 10.1186/s12859-024-05868-6

**Published:** 2024-08-07

**Authors:** Katharina Munk, Daria Ilina, Lisa Ziemba, Günter Brader, Eva M. Molin

**Affiliations:** Center for Health & Bioresources, AIT Austrian Institute of Technology, Konrad‑Lorenz‑Straße 24, 3430 Tulln, Austria

**Correction: BMC Bioinformatics (2024) 25:93** 10.1186/s12859-024-05719-4

Following the publication of the original article [1], the authors identified that uploading the data into the Shiny application Holomics was not possible. Additional file 1 and the description of how to prepare the data have been updated.

The original article [1] has been corrected.

### Supplementary Information

**Additional file 1**. The additional file (Additional file 1.zip) is a compressed folder containing four.csv files. Table S1: Targeted metabolite data, Table S2: Microbiomics ASV count table resulting from 16S amplicon sequencing, Table S3: Microbiomics ASV count table resulting from ITS amplicon sequencing, Table S4: Transcriptomics read count table (transposed format), and Table S5: Labels and class information including color code of the analyzed samples. Besides of being the data source for the present case study, these data tables can be used as test datasets after removal of the table header (first line). We highly recommend opening the files in a text editor of your choice and remove the headers there. When doing this step in Excel an error may occur.
